# Dendritic Cells: Immune Response in Infectious Diseases and Autoimmunity

**DOI:** 10.1155/2020/2948525

**Published:** 2020-03-20

**Authors:** Renata Sesti-Costa, Pedro Manoel Mendes de Moraes-Vieira, Luisa Cervantes-Barragan

**Affiliations:** ^1^Hematology and Hemotherapy Center, UNICAMP, Campinas, SP, Brazil Rua Carlos Chagas, 480. 13083-878; ^2^Department of Genetics, Evolution, Microbiology and Immunology, Institute of Biology, University of Campinas, Campinas, Brazil; ^3^Department of Microbiology and Immunology, Emory University, School of Medicine, Atlanta, GA, USA

Dendritic cells (DCs) are the sentinels of the immune system. They sense, process, and present antigens to T lymphocytes orchestrating the immune response. The discovery of DCs granted the Nobel Prize for Ralph Steinman, who noticed the presence of rare cells in a culture of mice adherent cells with a distinct stellate morphology [1]. Years later, these cells were shown to express high amounts of major histocompatibility complex class II (MHC-II) molecules, and, even though in very low numbers, they revealed to be the cells responsible for activation and stimulation of naive T lymphocyte [2]. DCs not only activate and subsequently polarize lymphocyte but also can have a tolerogenic role, which is dependent on the factors derived from the surrounded microenvironment [3] and is crucial for the outcome of infectious diseases and autoimmunity by protecting the body from immune-mediated tissue damage. This tolerogenic property is also a target of manipulation by tumor cells to evade the immune response. Thus, understanding the precise regulation of DC function mediated by the different stimulus, such as cytokines and other mediators, remains the goal of numerous studies aiming at skewing T lymphocytes polarization in vaccination protocols for both cancer and infectious diseases.

Since the discovery of DCs, they have been extensively studied in several contexts, from immunity to pathogens and tumors to the induction of tolerance or an immune response to transplants, to self, and more recently to microbiota and dietary antigens, with new discoveries happening constantly. Recent advances in the field have pointed at new DC function as well as new DC subsets with specific transcriptomes [4]. DCs are a heterogeneous group of cells that perform different functions in the immune response. Although the specific phenotype of DCs may differ depending on tissue location and inflammatory context, there are four main groups of DCs identified in both mice and humans, (1) type 1 and (2) type 2 conventional DCs, which have high capacity of processing and presenting antigens to T lymphocytes, (3) plasmacytoid DCs (pDCs), which are the major IFN-I producers, and (4) inflammatory DCs, which are monocyte-derived cells in an inflammatory context that produce inflammatory cytokines [5–7]. Recently, a single-cell RNA-seq study reclassified human DCs into six transcriptionally different subpopulations, two novel types in addition to those aforementioned. One of them is related to pDCs, with the potential to activate T cell, and another subdivision within the class of cDCs1 [8].

The mechanisms of DC development either from progenitors of bone marrow or from circulating monocytes have also gained much knowledge in the past years, enlightening the signaling pathways activated in the rising of the distinct DC subsets. They are either GM-CSF-mediated STAT5 phosphorylation- or FLT3-L-mediated STAT3 phosphorylation-dependent pathways. Moreover, several transcription factors have been identified as subtype specific for the development of DCs in mouse models [9, 10], such that changes in the cytokine microenvironment and in the transcription factors expressed in dendritic cell progenitors are able to switch DC subset and may influence the outcome of the immune response. Thereby, this complexity makes evident that the study of the development and function of DCs still has much to reveal. The expansion of our understanding of the roles of these unique cells in different scenarios and their underlying mechanisms is crucial for the design of new and specific therapeutic approaches.

This special issue presents a collection of original research articles that unveil the role of dendritic cells, their cellular interactions, and the molecular mechanisms and signaling pathways involved in clinical and experimental models of infections, inflammatory disease, and cancer. First, Azevedo-Santos et al. investigated the mechanisms of the tolerogenic role of DCs from breast tumor patients. In this patients' cohort, they corroborated previous data by demonstrating that monocyte-derived DCs (mo-DCs) from cancer patients are less mature, have a decreased ability to stimulate T lymphocyte proliferation, and produce the anti-inflammatory cytokine IL-10. In addition, they showed that completely differentiated DCs from patients presented the same phenotype as DCs from healthy donors when cocultured with breast cancer-derived cell lines. Nevertheless, monocytes isolated from cancer patients expressed less GM-CSF and IL-4 receptors than monocytes isolated from healthy donors, which may inhibit the differentiation of inflammatory DCs from circulating monocytes. They correlated the presence of the heat shock protein 27 (Hsp27), which is involved in tumor cell proliferation and invasion, with the tolerogenic profile of DCs. In addition to previous data that showed Hsp27 expression in tumor cells, they also revealed that mo-DCs derived from cancer express higher levels of Hsp70. The direct involvement of DC-expressed Hsp27 with the anti-inflammatory response to the tumor still needs clarification.

The maturation state of DCs was the aim of investigation of two articles that show its role in different infectious and inflammatory diseases. In the first article, Islam et al. observed a higher frequency of circulating CD83^+^ DCs in a mouse model of herpes simplex virus-1- (HSV-1-) induced Behçet's disease, when comparing asymptomatic and symptomatic mice. By performing a set of experiments blocking CD83 *in vivo* by different techniques, they revealed an improvement on the severity score associated with reduced frequency of CD83^+^ DCs and downregulation of IL-17 serum levels, which demonstrates the involvement of DCs in the pathology of the disease. Interestingly, ablation of CD83 blockade worsened the symptoms, whereas reestablishment of CD83 inhibition with siRNA treatment, even in the late stage of the disease, restored the beneficial effects and indicates that CD83 is a good candidate for novel therapeutic approaches in Behçet's disease. In the second article, Helmin-Basa et al. detected increased circulating CD83-expressing cDCs in Helicobacter *pylori*-infected children compared to healthy controls, although the percentages and total number of DCs were similar. The induction of circulating cDC maturation was associated with cDC infiltration in the gastric lamina propria, albeit infiltrating DCs did not express CD83, indicating a more immature profile of DCs in the infected site, which might induce tolerance instead of immunity to local antigens. This set of articles highlighted the importance of DC maturation not only for immunity against HSV-1 and *H. pylori* infections but also for the immune response-driven pathology.

Another article within this special issue by Barbosa et al. emphasized how DC responses can differ depending on the strain of the pathogen involved, even if the strains are closely related, pointing out that DC functions are responsible for the outcome of the infection. They showed that the Mexican *Trypanosoma cruzi* strain Ninoa infected mo-DC more effectively than the Brazilian strain CL-Brener or even another Mexican strain INC5, all three classified as the same genotype subgroup. mo-DCs infected with Ninoa produced more TNF-*α* and IL-10 than mo-DCs infected with the other strains. The response varied also if the mo-DCs were derived from either BALB/c or C56BL/6 mice, evidencing that phylogenetically close pathogens use different pathways for the modulation of DC function, which affects the course of infection and should be taken into consideration for treatment.

The last set of articles explored the interactions of DCs with other cells and their effects on the immune response to infection in different models. Zhao et al. elegantly showed by both *in vitro* and *in vivo* approaches that DCs play the role of intermediary between invariant natural killer T cells (iNKT) and NK cells during *Chlamydia pneumoniae* infection in mice, which indirectly helps bacterial clearance. They revealed a role for iNKT cells in the regulation of the protective IFN-*γ*-producing CD27^high^ NK cells by infecting iNKT cell-deficient mice, which displayed changes in NK subsets and an inhibition of NK activation and IFN-*γ* production. Previous studies demonstrated the role of DCs in NK cell activation and cytokine production; however, *in vitro*, coculture of NK cells with DCs from iNKT deficient mice resulted in reduced expression of CD69 and CD25 and impaired of IFN-*γ* production by NK cells compared to coculture with WT DCs. Interestingly, adoptive transfer of DCs from either WT or iNKT-deficient mice to infected mice resulted in the same effect observed *in vitro*, demonstrating that iNKT interaction with DCs allows them to modulate NK cell responses. Loss et al. also explored the cellular networks important against bacterial invasion. They studied the crosstalk between porcine mo-DCs and intestinal epithelial cells (IEC). Using *in vitro* assays, they showed that the enteropathogenic bacteria *Escherichia coli* but not the probiotic bacteria *Enterococcus faecium* modulated NLRP3-dependent inflammasome signaling. Contact-dependent communication between mo-DCs and IECs increased inflammasome activation in IECs, whereas it weakened IL-1*β* production by DCs. Altogether, these data illustrate the complexity of cellular interactions necessary to orchestrate the precisely effective immune response by different species to fight against a pathogen. We hope you enjoy the reading of this special issue and that it may give you new insights into the biology of DCs, this unique cell with varied functions in diverse context.
